# Trimethyltin(IV) Bearing 3-(4-Methyl-2-oxoquinolin-1(2H)-yl)propanoate Causes Lipid Peroxidation-Mediated Autophagic Cell Death in Human Melanoma A375 Cells

**DOI:** 10.3390/ph17030372

**Published:** 2024-03-14

**Authors:** Marijana P. Kasalović, Dušan Dimić, Sanja Jelača, Danijela Maksimović-Ivanić, Sanja Mijatović, Bojana B. Zmejkovski, Simon H. F. Schreiner, Tobias Rüffer, Nebojša Đ. Pantelić, Goran N. Kaluđerović

**Affiliations:** 1 Department of Engineering and Natural Sciences, University of Applied Sciences Merseburg, Eberhard-Leibnitz-Straße 2, 06217 Merseburg, Germany; marijana.kasalovic@pmf.kg.ac.rs; 2 Department of Chemistry, Faculty of Science, University of Kragujevac, Radoja Domanovića 12, 34000 Kragujevac, Serbia; 3 Faculty of Physical Chemistry, University of Belgrade, Studentski trg 12-16, 11000 Belgrade, Serbia; ddimic@ffh.bg.ac.rs; 4 Department of Immunology, Institute for Biological Research, “Siniša Stanković”—National Institute of the Republic of Serbia, University of Belgrade, Bulevar despota Stefana 142, 11108 Belgrade, Serbia; sanja.jelaca@ibiss.bg.ac.rs (S.J.); nelamax@ibiss.bg.ac.rs (D.M.-I.); sanjamama@ibiss.bg.ac.rs (S.M.); 5 Department of Chemistry, Institute of Chemistry, Technology and Metallurgy—National Institute of the Republic of Serbia, University of Belgrade, Studenski trg 12-16, 11000 Belgrade, Serbia; bojana.zmejkovski@ihtm.bg.ac.rs; 6 Institute of Chemistry, Chemnitz University of Technology, Straße der Nationen 62, D-09111 Chemnitz, Germany; simon.schreiner@chemie.tu-chemnitz.de (S.H.F.S.); tobias.rueffer@chemie.tu-chemnitz.de (T.R.); 7 Department of Chemistry and Biochemistry, Faculty of Agriculture, University of Belgrade, Nemanjina 6, 11080 Belgrade, Serbia

**Keywords:** organotin(IV) complexes, trimethyltin(IV) compound, BSA, DFT, molecular docking, in vitro anticancer activity, ROS/RNS

## Abstract

A novel trimethyltin(IV) complex (**Me_3_SnL**), derived from 3-(4-methyl-2-oxoquinolin-1(2H)-yl)propanoate ligand, has been synthesized and characterized by elemental microanalysis, UV/Vis spectrophotometry, FT-IR and multinuclear (^1^H, ^13^C and ^119^Sn) NMR spectroscopies. Furthermore, the structure of the ligand precursor **HL** was solved using SC-XRD (single-crystal X-ray diffraction). The prediction of UV/Vis and NMR spectra by quantum-chemical methods was performed and compared to experimental findings. The protein binding affinity of **Me_3_SnL** towards BSA was determined by spectrofluorometric titration and subsequent molecular docking simulations. **Me_3_SnL** has been evaluated for its in vitro anticancer activity against three human cell lines, MCF-7 (breast adenocarcinoma), A375 (melanoma) and HCT116 (colorectal carcinoma), and three mouse tumor cell lines, 4T1 (breast carcinoma), B16 (melanoma) and CT26 (colon carcinoma), using MTT and CV assays. The strong inhibition of A375 cell proliferation, ROS/RNS upregulation and robust lipid peroxidation lead to autophagic cell death upon treatment with **Me_3_SnL**.

## 1. Introduction

Cancer is a medical term for a group of malignant diseases that are described by the development and uncontrolled growth of abnormal cells, followed by the disintegration of normal body tissue [1,2]. The main weapons for the treatment of cancer are chemotherapy, radiotherapy and surgery [3]. In chemotherapy, cisplatin was successfully introduced in cancer therapy by the United States Food and Drug Administration (FDA) in 1978, and within its platinum(II)-analogues, it remains the most commonly used chemotherapeutic agent for the treatment of ovarian, testicular, breast, kidney and other cancers [4,5]. The effective clinical utilization of cisplatin- and platinum(II)-based drugs is limited by the high toxicity, resistance and severe side effects that appear after treatment [6,7]. The necessity for the development of new chemotherapeutic agents is of great importance, and the research nowadays is shifted to the development of non-platinum transition metal complexes (Sn, Ti, Ga, etc.) and organometallic compounds [8,9].

Organotin(IV) compounds have received considerable attention in recent years due to their commercial and biomedical applications [10]. Regarding to their availability, many organotin(IV) compounds have been investigated for their biological activity, in order to find new medical applications [11]. Reports have shown that these compounds display antimicrobial, antimalarial, anti-HCV, antidiabetic, antileishmanial, antiproliferative and antitumor properties. With Sn(II) and Sn(IV) oxidation states, tin might be considered as a potential replacement for cisplatin, and comprehensive literature can provide certain tracks for the future synthesis of novel organotin(IV) complexes as potential anticancer agents [12]. According to these reports, the biological activity of organotin(IV) compounds is related to the number and nature of organic substituents bound directly to the tin atom, but the coordinated ligand also has an influence [13,14]. Furthermore, a correlation between cytotoxicity and lipophilicity was observed. The triorganotin(IV) compounds exert the best cytotoxic effect, compared to their diorganotin(IV) and monoorganotin(IV) analogues. The best cytotoxic activity of triorganotin(IV) compounds is attributed to their highest lipophilicity, the availability of coordination positions at the tin center and their affinity to proteins [3,15,16,17]. The organotin(IV) carboxylates have shown the highest anticancer activity in comparison with organotin(IV) thiolates and dithiocarbamates [18]. In terms of advantages over cisplatin, organotin(IV) compounds are cheaper and widely available, and they are less toxic and induce apoptosis at low doses so the cells do not develop resistance to them [13,19,20]. The low solubility of organotin(IV) compounds in water could affect in vivo experiments. In view of the high cytotoxicity, it is therefore important to synthesize new organotin(IV) compounds with a higher water solubility [8].

As mentioned above, ligands also have an impact on anticancer activity, and the literature suggests that a ligand must provide a relatively stable ligand–tin bond and its slow hydrolytic decomposition [21]. Quinolones, antibiotics with good oral absorption, safety and excellent availability, are among the most desirable heterocycle skeletons in drug research [22,23]. They have a privileged structure for variety modifications, and many quinolone derivatives are used in medicinal chemistry due to their biological activity, such as antibacterial, antitubercular, antimalarial, anti-HIV, anti-HCV, anticancer, etc. [22,24,25]. New strategies for designing metal complexes with biologically active drugs include quinolones as ligands. Among organotin(IV) complexes with quinolones as ligands, only a few organotin(IV) complexes with fluoroquinolones have been reported [21,26]. Although organotin(IV)-quinolone coordinated systems have great potential, not many studies have investigated their anticancer activity, nor the mechanism of their action.

In view of all the benefits that could be achieved by the coordination of organotin(IV) moiety with quinolones, the trimethyltin(IV) complex with 3-(4-methyl-2-oxoquinolin-1(2H)-yl)propanoate, **Me_3_SnL**, was prepared in order to examine its anticancer potential and compare the results with similar organotin(IV) compounds [27]. The quantum-chemical analysis and spectral prediction confirmed the proposed structure based on multinuclear NMR and IR spectroscopies as well as UV/Vis spectrophotometry. The interactions with the transport protein were examined by spectrofluorometric titration and molecular docking simulations. The in vitro anticancer activity of the newly synthesized trimethyltin(IV) complex **Me_3_SnL** was determined using MTT and CV assays against the following tumor cell lines: human breast adenocarcinoma (MCF-7), human colorectal carcinoma (HCT116), human melanoma (A375), mouse breast carcinoma (4T1), mouse colon carcinoma (CT26) and mouse melanoma (B16). Furthermore, the mode of action of **Me_3_SnL** on A375 cells, such as caspase activity, apoptosis, proliferation, autophagy, ROS/RNS production and lipid peroxidation detection, was evaluated by flow cytometry.

## 2. Results and Discussion

### 2.1. SC-XRD Analysis, Structure Optimization and Hirshfeld Surface Analysis of HL

#### 2.1.1. Molecular Structure of HL

**Figure 1:**
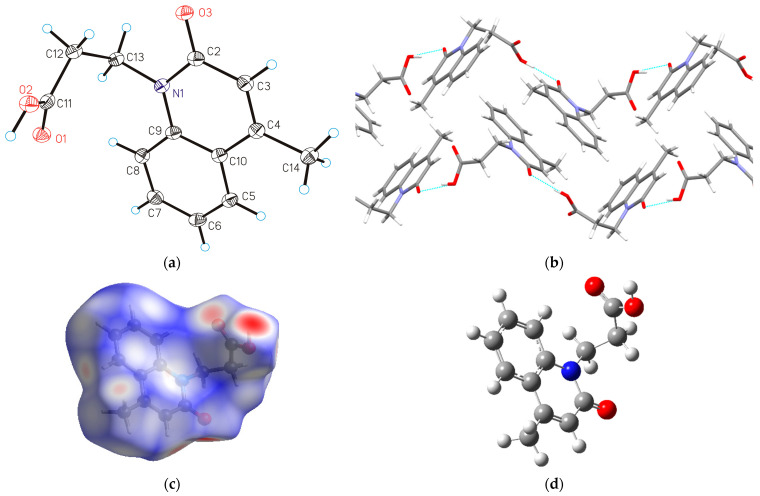
(**a**) ORTEP (50% probability ellipsoids) perspective view of the molecular structure of the ligand precursor **HL** with the atomic numbering scheme; (**b**) Mercury view of packing and hydrogen bonding (propagation along *z*-axis); (**c**) Hirshfeld surface and (**d**) optimized structure (at B3LYP-D3BJ/6-311++G(d,p) level of theory) of **HL**. (Hydrogen-white, carbon-gray, nitrogen-blue, oxygen-red).

3-(4-Methyl-2-oxoquinolin-1(2H)-yl)propanoic acid (**HL**) crystallizes in the monoclinic *P2_1_/c* space group with *Z* = 4 (Table S1). The perspective view of the molecular structure is shown in Figure 1a, while the selected bond lengths and angles are presented in Table S2. Molecular **HL** is built from a quinolone bicyclic core, the propionic group bonded to the nitrogen atom N1 and the methyl substituent in position 4 (C6) of the quinolone ring. As expected, **HL** is approximately planar, with small deviations indicated by the torsion angles C13–N1–C4–C5 = –2.85 (17)° and C4–N1–C13–C8 = 1.76 (18)°. The intermolecular hydrogen bond O2−H1⋯O3 stabilizes the crystal structure forming polymeric chains (O2−H1⋯O3: 2.628 (1) Å; O2–H1⋯O3: 165 (3)°, Figure 1b). The chains assemble through π-σ (2.684 Å) as well as π-π -stacking interactions with sliding between the interacting moieties (distance between two parallel quinolone moieties 3.386 Å, Figure S1) in the overall 3D network structure.

#### 2.1.2. Geometry Optimization of HL

**Scheme 1:**
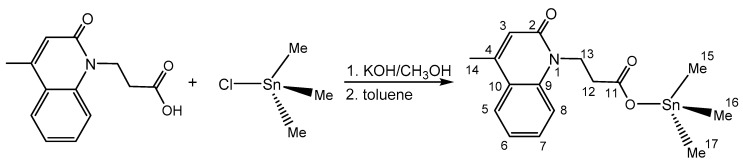
Synthesis of the trimethyltin(IV) complex **Me_3_SnL** (with numbering of the carbon atoms).

The crystallographic structure of **HL** served as a starting configuration for the optimization at the B3LYP-D3BJ/6-311++G(d,p) level of theory. The experimental and optimized bond lengths and angles are listed in Tables S3 and S4, following the atom numeration from Scheme 1. These two sets of data were compared by calculating the correlation coefficients and mean absolute error (MAE) [28].

When experimental and theoretical bond lengths are compared, it can be concluded that the optimized ones reproduce the crystallographic data well. The correlation coefficient between these two sets is 0.99, with an MAE of 0.008 Å, the order of the experimental error. The most significant differences were found for bonds containing carbon and electronegative atoms, such as C−N, C−O and C=O. Larger discrepancies were calculated for the bond angles. The experimental values correlate well with the optimized ones, with a correlation coefficient of 0.97 and an MAE of 0.6°. Angles within the aliphatic chain differ from the experimental structure between 0.1 and 2.2°. These differences can be expected, bearing in mind that the optimization was performed for the isolated structure in a vacuum, while in the crystal structure, different intermolecular interactions limit the position of the aliphatic chain, as shown in the previous section. Nevertheless, these results verify the applicability of the selected level of theory.

Different intramolecular interactions are responsible for stabilizing the molecular structure, some of which are listed in Table S5. These energies were calculated through the second-order perturbation theory approach, as implemented in the Gaussian program package. The most numerous interactions within the oxoquinoline moiety are formed between carbon–carbon bonds, denoted as π(C−C)→π*(C−C), with stabilization energies between 72 and 98 kJ mol^−1^. The coplanarity of the methyl group with the rest of the ring structure is stabilized through the interaction between π(C−C) and σ*(C4−C14), with an energy of 75 kJ mol^−1^. The presence of an oxygen atom directly attached to the ring structure additionally stabilizes the overall structure through several interactions. One of these interactions can be denoted as π(C−C)→π*(C−O) (101 kJ mol^−1^). In the second type of interaction, an oxygen atom is a donor through LP(O)→π*(C2−C3) and LP(O)→(N−C2) interactions, with stabilization energies of 61 and 100 kJ mol^−1^, respectively. Nitrogen atoms also influence the stability by interacting with neighboring carbon–carbon and carbon–oxygen bonds (141 and 247 kJ mol^−1^). The stabilization interactions within carboxylic acid can be denoted as LP(O)→π*(C−O) (134 kJ mol^−1^). These oxygen atoms stabilize the neighboring C11−C12 bond. It should be noted that bonds of the aliphatic part are not included in these interactions, which allows for the relative flexibility of this part of the molecule, as previously discussed. In the following section, intermolecular interactions governing complex stability are discussed, and differences between free and bound ligands are outlined. 

#### 2.1.3. Hirshfeld Surface Analysis

The Hirshfeld surface of **HL** is presented in Figure 1c, while fingerprint plots for the most important contacts are shown in Supplementary Material as Figure S2. As the structure of **HL** consists of oxoquinoline and carboxylic groups, it can be expected that polar groups are the ones that form the strongest interactions with the surrounding units. This assumption is verified by the surface color, as the red spots are close to carboxylic acid and carbonyl oxygen. The most numerous contacts can be denoted as H⋯H, and they account for 43.7%. This result is expected, as hydrogen atoms are part of oxoquinoline and methyl groups. The interactions between the oxygen atom of carbonyl, the carboxyl group and the hydrogen atom are present with 27.8%, similar to the compounds in reference [29]. Conventional hydrogen bonds are included in this group [30]. Weak hydrogen bonds, formed between carbon atoms and hydrogen (H⋯C, 18.4%), denote various interactions such as those between positively charged hydrogen atoms and the π electron cloud of the aromatic part [31]. Interactions between two carbon atoms are very limited (5.6%) due to the presence of adjacent hydrogen and oxygen atoms. The same applies to the interactions represented by H⋯N (1.9%). The rest of the interactions are present in much lower percentages (O⋯C (1.4%), O⋯O (0.7%), and N⋯C (0.5%)).

**Figure 2:**
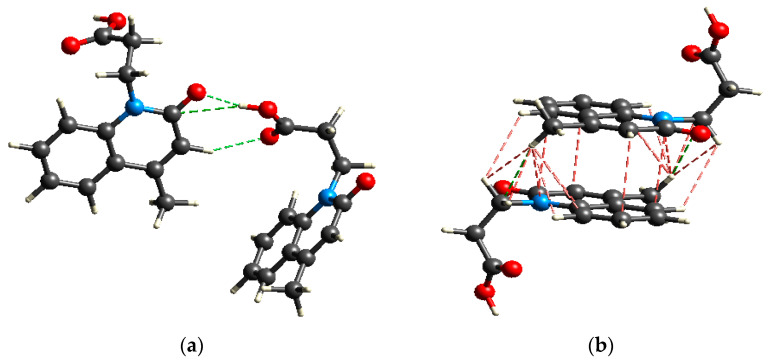

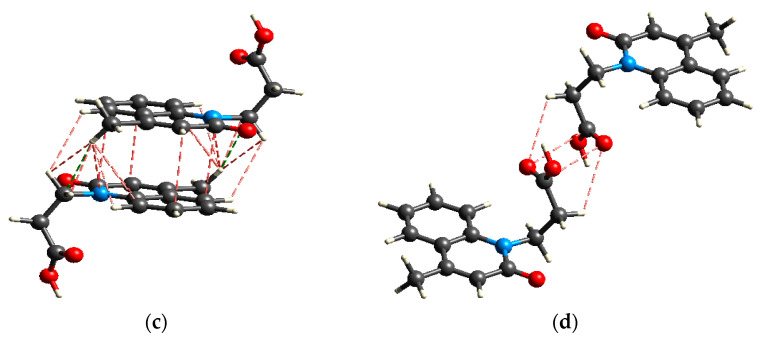
Isolated dimer structures in the crystals of **HL** (at B3LYP/6-31G(d,p) level of theory): dimer 1 (**a**), 2 (**b**), 3 (**c**) and 4 (**d**). (Hydrogen-white, carbon-gray, nitrogen-blue, oxygen-red, dotted lines-different interactions between units).

The values of interaction energies for the specific positions of two ligand molecules are obtained at the B3LYP/6-31G(d,p) level of theory, which proved sufficient for the relative comparison of the interaction strength. The trend in obtained energies is the same as that obtained by employing more costly levels of theory [31,32]. Figure 2 presents dimers with characteristic interactions formed between them. The first dimer allows for the formation of a strong hydrogen bond between the carboxyl group of one monomer and the carbonyl oxygen of the other, with an interaction energy of −56.9 kJ mol^−1^. The interactions also include weak hydrogen bonds denoted as O−H⋯C and C−H⋯O. The interaction energy between dimers in the second structure is lower, −45.5 kJ mol^−1^. In this structure, monomers are ideally positioned for the weak stacking interactions through contacts C⋯C and C⋯H. Weak hydrogen bonds, C−H⋯O, are also present. In the structure of the third dimer, two molecules are positioned perpendicularly with an interaction energy of −30.8 kJ mol^−1^. Within this dimer, interactions include only weak hydrogen bonds such as C−H⋯O and C−H⋯C (C−H⋯π). The lowest interaction energy was obtained for dimer 4 (−13.4 kJ mol^−1^), in which only O⋯O and H⋯O contacts are present. The relative positions of two carboxyl groups limit the formation of stronger hydrogen bonds. This type of analysis is essential when novel materials with desired properties in a solid state are prepared [32,33]. 

### 2.2. Me_3_SnL: Synthesis, Experimental and In Silico Characterization

#### 2.2.1. Chemistry and DFT

**Figure 3:**
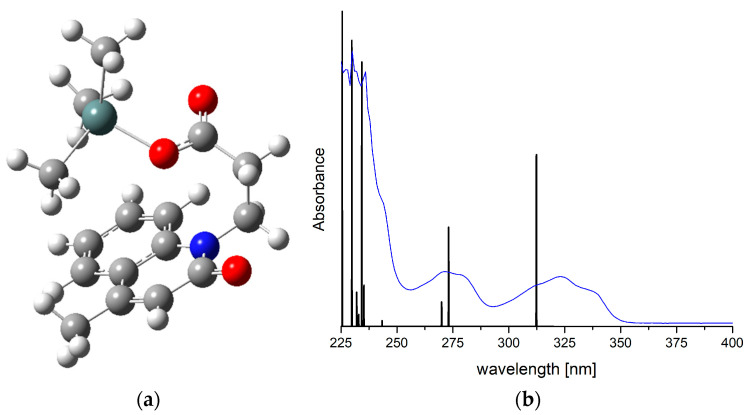
(**a**) Optimized structure (at the B3LYP-D3BJ/6-311++G(d,p)(H,C,N,O)/Def2-TZVP(Sn) level of theory; atom representation: hydrogen-white, carbon-gray, nitrogen-blue, oxygen-red, tin-teal) and (**b**) experimental/theoretical UV/Vis spectra in water of **Me_3_SnL** (experimental spectrum—blue line, theoretical electronic transitions—black bars).

The trimethyltin(IV) complex was synthesized by reacting a toluene solution of trimethyltin(IV) chloride and the potassium salt of **HL** in a molar ratio of 1:1 (Scheme 1). The resulting complex is soluble in chloroform, dichloromethane, methanol, toluene and dimethylsulfoxide but insoluble in water. The structure of the synthesized compound was determined by FT-IR and multinuclear NMR spectroscopy. The purity of **Me_3_SnL** was confirmed by elemental microanalysis. The proposed structure of **Me_3_SnL** was confirmed by comparing theoretical and experimental NMR and UV/Vis spectra, as all trials intended to crystallize this compound were not successful. The molecular structure of **Me_3_SnL** was geometry-optimized at the B3LYP-D3BJ/6-311++G(d,p)(H,C,N,O)/Def2-TZVP(Sn) level of theory (Figure 3a). The same functional was used in the literature to describe the structure and assign the spectra of other organotin compounds [34,35,36,37].

The most important stabilization interactions within the complex are presented in Table S4. Only interactions including tin(IV) and surrounding atoms are discussed. The strongest interaction is formed between the lone pair of carboxylic oxygen and empty orbitals of tin(IV), LP(O)→LP*(Sn) (694 kJ mol^−1^). This lone pair additionally stabilizes bonds formed between tin(IV) and carbon atoms of methyl groups (LP(O)→σ*(Sn−C), 46 kJ mol^−1^). The carbon–oxygen bond of the carboxylic group acts as a donor for several interactions with neighboring Sn−C bonds (between 43 and 291 kJ mol^−1^). The electron donation also occurs from this bond to tin (π(C−O)→LP*(Sn), 79 kJ mol^−1^). Methyl groups surrounding tin(IV) are essential for the stabilization of the complex, as there are a multitude of weak interactions of type σ(C−H)→LP*(Sn) (24 kJ mol^−1^). Carbon–hydrogen bonds of methyl groups also stabilize carbon–tin bonds with an energy of 67 kJ mol^−1^. Interactions between C−Sn bonds in which carbon atoms originate from different methyl groups are present with stabilization energies of 53 kJ mol^−1^.

#### 2.2.2. UV/Vis Spectrophotometry and IR Spectroscopy

The UV/Vis spectrum of **Me_3_SnL** was recorded for a solution obtained after the dilution of the stock solution in DMSO (10^−3^ M) with water to a final concentration of 10^−4^ M. The spectra were recorded between 200 and 500 nm. At the same time, the theoretical one was calculated for the structure reoptimized in water by employing TD-DFT methodology, as it was assumed that the experimental amount of DMSO in the solution was negligible. The experimental and theoretical UV/Vis spectra are given in Figure 3b. The experimental UV/Vis spectrum consists of three broad peaks at 323, 274 and 235 nm. In the theoretical spectrum, the HOMO→LUMO (93%) transition is located at 312 nm, with an oscillator strength of 0.1307. The difference between the experimental and theoretical wavelength is 11 nm, which is acceptable, bearing in mind that the solvent effect through specific solvent–solute interaction can be responsible for the shift [38]. An explicit solvent model would probably decrease this difference. The second peak in the experimental spectrum can be assigned to the HOMO-1→LUMO (68%) transition, with an oscillator strength of 0.0185 located at 274 nm in the theoretical spectrum. There are three high-intensity peaks in the theoretical spectrum that represent an intense experimental peak around 235 nm. These peaks are located at 235 (HOMO→LUMO+1, 41%), 232 (HOMO→LUMO+3, 55%) and 226 nm (HOMO→LUMO+4, 39%), with oscillator strengths above 0.200. It should be mentioned that the relative intensities of the maxima in the UV/Vis spectrum, besides positions, were well reproduced, proving that the optimized structure can be considered a good representation of the experimental one. 

The vibrations in the FT-IR spectrum (Figure S3) of **Me_3_SnL** were assigned by comparison with those of the ligand precursor (Figure S4). The formation of trimethyltin(IV) bearing carboxylate ligand (**L^−^**) was confirmed by the disappearance of the strong ν(COOH) absorption at 1728 cm^−1^ and the appearance of two new bands at 1574 and 1371 cm^−1^ related to the asymmetric [ν_asym_(COO^−^)] and symmetric [ν_sym_ (COO^−^)] vibrations (Figure S1). A difference between the asymmetric and symmetric vibrations of the carboxylic group, Δν (Δν = ν_asym_ − ν_sym_), which lies over 200 cm^−1^, indicates a monodentate coordination mode of the carboxylic group in a solid state. Besides the vibration at 546 cm^−1^ assigned to ν(Sn−C) at 546 cm^−1^, the appearance of an additional band at 447 cm^−1^, ν(Sn−O), further supports the bonding of the carboxylic oxygen atom to the trimethyltin(IV) moiety [39,40].

#### 2.2.3. Multinuclear NMR Spectroscopy

The ^1^H, ^13^C and ^119^Sn NMR spectra are shown in Figure S5. The assignments of proton and carbon chemical shifts of the compound **Me_3_SnL** were achieved by comparing with the NMR data obtained from an appropriate ligand precursor deprotonated in the same solvent as the NMR measurements of **Me_3_SnL** were performed (CDCl_3_ solution). The resonances of the methyl groups in **Me_3_SnL** were determined by examining the chemical shift values and multiplicity patterns. Furthermore, the coupling constants ^1^*J*(^119^Sn, ^13^C) and ^2^*J*(^119^Sn, ^1^H) and the correlations of their magnitudes with coordination geometry are important parameters for the structural elucidation of the organotin(IV) compounds [41]. For tetracoordinated tetrahedral trimethyltin(IV) compounds, the coupling constant ^1^*J*(^119^Sn, ^13^C) values are predicted to be less than 400 Hz, and the coupling constant ^2^*J*(^119^Sn, ^1^H) values are less than 59 Hz. For pentacoordinated ^1^*J*(^119^Sn, ^13^C) trimethytin(IV) compounds, the values fall in the range of 450–670 Hz, and for ^2^*J*(^119^Sn, ^1^H), the values fall in the range of 65–80 Hz. The values for ^1^*J*(^119^Sn, ^13^C) and the ^2^*J*(^119^Sn, ^1^H) for hexacoordinated organotin(IV) complexes are larger than 670 and 83 Hz, respectively [14,42,43]. 

The ^1^H NMR spectral data for aromatic protons that originate from the quinolone skeleton are observed in the range of 6.58–7.72 ppm. The resonance of the methylene protons in the α-position to the carboxylic group is shifted to a higher value for 0.17 ppm in comparison with the deprotonated ligand, and this indicates the coordination through the close carboxylic group [27]. The methyl protons of **Me_3_SnL** appear as a sharp singlet at 0.58 ppm, along with the clearly visible satellites, with small intensities, providing a ^2^*J*(^119^Sn, ^1^H) coupling value of 58/56 Hz. The Lockhart and Mander’s Equation (1) is as follows:

*θ*(C-Sn-C) = 0.0161|^2^*J*(Sn-H)|^2^ − 1.32|^2^*J* (Sn-H)| + 133.4 (1)

It was applied to the coupling constant ^2^*J*(^119^Sn-^1^H) to calculate the angle between C–Sn–C and confirm the tetrahedral geometry around the tin atom in a non-polar solution state. The calculated C–Sn–C angle of 111° implies a tetrahedral geometry, since the C–Sn–C angle for tetracoordinated organotin(IV) compounds is reported to be *θ* ≤ 112° [14,44,45].

The experimental and theoretical ^1^H and ^13^C NMR chemical shifts are shown in Table 1. The calculated values were systematically overestimated, and the correction coefficients were determined from the dependency between the experimental and theoretical data.

In the case of resonances in the ^1^H NMR spectrum, these two sets of chemical shifts show a high resemblance with a correlation coefficient of 0.998 and an MAE value of 0.19 ppm. The lowest chemical shifts were calculated for protons of methyl groups. Upon coordination, the chemical shifts of the hydrogens from the propionic moiety are shielded (for example, 2.73 ppm in **Me_3_SnL** and 2.56 ppm in **L^−^**). This is a consequence of the electron density shift towards the central metal ion, which aligns with the previous analysis from Section 3.1. Protons from the quinolone moiety have chemical shifts between 6.58 and 7.72 in the experimental spectrum and between 6.45 and 6.64 ppm in the theoretical spectrum. The resonances of these protons are almost identical to those of the free ligand, which proves the assumption that this part of the ligand is not directly affected by the interactions with tin(IV).

In the ^13^C NMR spectrum of **Me_3_SnL**, for aromatic carbon and carbonyl carbon atoms, no significant difference in the resonances compared to **HL** or **L^−^** was observed. The resonance of the methyl carbon atoms from the **Me_3_Sn** moiety was assigned in comparison with other trimethyltin(IV) carboxylates [14,40], combined with the coupling constant ^1^*J*(^119^Sn, ^13^C). This chemical shift was observed at −2.75 ppm, along with the small satellites which give the coupling constant ^1^*J*(^119^Sn, ^13^C) a value of 400/384 Hz. The Lockhart’s Equation (2) is as follows:

^1^*J*(^119^Sn, ^13^C) = 11.4*θ* − 875 (2)

By applying it on the ^1^*J*(^119^Sn, ^13^C) coupling constant, the calculated angle C–Sn–C of 111.84° also confirms the tetrahedral geometry around the tin in non-polar solvents [42,44]. In the experimental ^13^C NMR spectrum, the resonance of the carboxylic carbon atom is located at 175.52 ppm, which is 0.32 ppm lower than in the L^−^. The difference in the theoretical spectrum is a bit lower, although a shift towards higher values is determined. Due to the low electronegativity of tin(IV), the chemical shifts of three methyl carbon atoms are found at −2.75 in the experimental and −0.39 ppm in the theoretical spectrum. The complex formation led to slight changes in the resonances of aliphatic carbon atoms, although these differences are less than 1.5 ppm. The same applies to carbon atoms of the quinolone part of the molecule. The final proof that the coordination of the carboxylic group is the difference between the resonances of the COO carbon atom in **Me_3_SnL** and **HL**/**L^−^**.

The geometry around the tin atom in **Me_3_SnL** was also confirmed by the ^119^Sn NMR study. In general, chemical shifts for tetracoordinated organotin(IV) complexes are found in the range from 200 to −60 ppm, those for pentacoordinated complexes are in the range from −90 to −190 ppm and those for hexacoordinated complexes are in the range from −210 to −400 ppm [45,46]. The ^119^Sn NMR spectrum for **Me_3_SnL** showed tin resonance at 130 ppm, suggesting tetrahedral geometry around the tin atom in non-polar solvents, and this is in agreement with other tetracoordinated trimethyltin(IV) carboxylates [45]. However, in DMSO-d_6_, the chemical shift for tin was found at −106 ppm, indicating trigonal bipyramidal geometry [47].

#### 2.2.4. Lipophilicity and Stability

In the Comprehensive Medicinal Chemistry database, the value of the partition coefficient (*logP*) between −0.4 and 5.6 qualifies the compound for clinical usage [48]. However, some reports indicate that compounds with a high lipophilic character (*logP* > 3) can cause hazard effects [49]. The trimethyltin(IV) complex **Me_3_SnL** has a *logP* value of 0.78; thus, this value qualifies the compound for potential further examinations of its biological activity and clinical utilization.

The stability of the complex **Me_3_SnL** was determined by recording the UV/Vis spectra of the compound in the water/DMSO solvent mixture immediately after dissolution and in intervals of 24, 48 and 72 h (Figure S6). As the absorbance maximum was shifted over time, we can conclude that the hydrolysis of the compound occurs.

### 2.3. Protein Binding Affinity of Me_3_SnL

**Figure 4:**
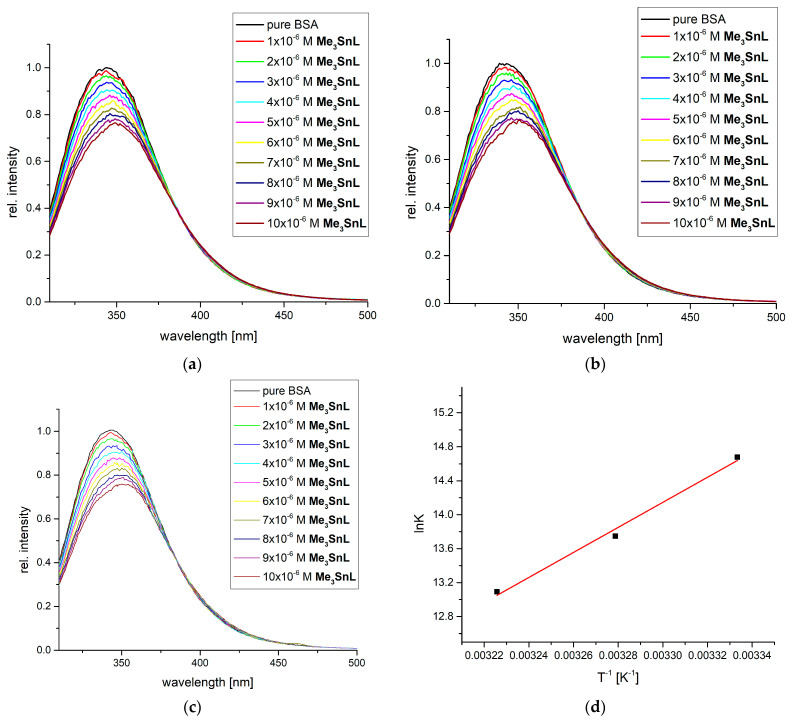
The fluorescence emission spectra of BSA before and after the addition of various amounts of **Me_3_SnL** at (**a**) 27, (**b**) 32 and (**c**) 37 °C, and (**d**) Van’t Hoff’s plot for the binding process between the complex and BSA.

The binding ability of compounds to transport proteins in blood is important for drug distribution, free concentration, metabolism and toxicity [50]. The mixture was irradiated by light with a wavelength of 295 nm, activating the fluorescence emission of tryptophan residues in positions 134 and 212. Their fluorescence intensity and position of maxima depend on interactions with compounds in the active pocket, as changes in the secondary structure of the protein influence the emission intensity. Figure 4 presents the fluorescence emission spectra of BSA before and after adding **Me_3_SnL** at three temperatures (27, 32 and 37 °C) to mimic the average body temperature.

As shown in Figure 4, the addition of **Me_3_SnL** to a solution of BSA led to a decrease in fluorescence emission intensity in a concentration-dependent manner. A slight bathochromic shift in the position of the emission of the maxima is additional proof that interactions between **Me_3_SnL** and BSA occur and that chemical surroundings in active positions were changed, as explained in reference [51]. The double-log Stern–Volmer equation was applied to examine this behavior. The linearity between the relative intensity decrease and the concentration of the quencher was proven by calculating the correlation coefficient (>0.997) [28]. The number of binding places is between 1.23 (37 °C) and 1.37 (27 °C), which signifies that one molecule of the complex was bound to one BSA molecule, as similarly obtained for diorganotin(IV) complexes with hydrazone Schiff bases [51]. The binding constants were 2.37 × 10^6^, 9.35 × 10^5^ and 4.85 × 10^5^ M^−1^ for measurements at 27, 32 and 37 °C, respectively. These values are comparable to those of diorganotin(IV) complexes with salicylaldehyde nicotinoyl hydrazone ligands [51]. The change in the enthalpy and entropy of binding was −122.7 kJ mol^−1^ and −287.4 J mol^−1^ K^−1^. The spontaneity of the binding process was shown, as the change in the Gibbs free energy of the binding was between −36.5 and −33.6 kJ mol^−1^ in the investigated temperature range. Upon binding, the system’s entropy decreases as the movement of the methyl groups and ligand is limited through interactions with surrounding amino acids in the active pocket. On the other hand, interactions between the protein and **Me_3_SnL** are stronger than the respective interactions between separated units, leading to the exothermicity of the process. The latter is a dominant contribution to the change in Gibbs free energy. These results can potentially explain the measured antitumor activity through the strong interaction formation between **Me_3_SnL** and proteins in cancer cells [52].

**Figure 5:**
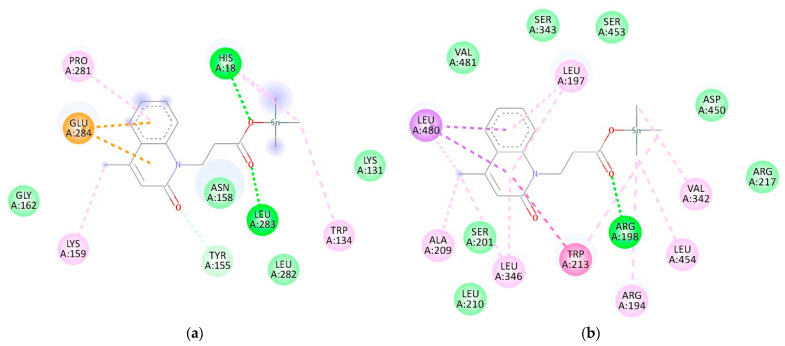
The most important interactions between **Me_3_SnL** and BSA in two active pockets around TRP134 (**a**) and TRP213 (**b**), as obtained in the molecular docking simulation (van der Waals and carbon hydrogen bonds—light green, conventional hydrogen bonds—dark green, π-alkyl—orange, π-π stacked—dark pink, alkyl and π-alkyl—light pink).

The molecular docking study between **Me_3_SnL** and BSA was performed to investigate the binding process at the atomic level. The spontaneity of this process was experimentally proven, and simulations were conducted in a way to determine the most probable binding position based on the similarity between experimental and theoretical values of thermodynamic parameters. The optimized structure at B3LYP-D3BJ/6-311++G(d,p)(H,C,N,O)/Def2-TZVP(Sn) was employed for the docking study. The obtained compound was positioned in the vicinity of two fluorescent amino acids, namely, TRP134 and TRP213, as those are the active positions that influence the fluorescent emission intensity the most. The interactions between amino acids and complex compounds are visualized in Figure 5, while the most important contributions to the binding energy are shown in Table S7. It is important to outline that **Me_3_SnL** binds spontaneously to both positions, which is in line with the experimental data. The binding energy in position TRP134 is −21.3 kJ mol^−1^, while in the second position, it is −29.0 kJ mol^−1^. In both active pockets, interactions with fluorescent amino acid were observed (Figure 5). Multiple interactions contribute to the values of the binding energies. In position one, there are two conventional hydrogen bonds formed between the carbonyl group of the complex and HIS18 and LEU282. Much weaker carbon hydrogen bonds include LEU282, GLY162, LYS131 and TYR155 (through carbonyl oxygen). Several other interactions, including π−alkyl and π−anion, were found. As presented, the main contribution to the binding energy comes from the van der Waals interactions, hydrogen bonds and desolvation (−27.8 kJ mol^−1^), while electrostatic interactions contribute 0.2 kJ mol^−1^. When experimental and theoretical binding energies are compared, it can be concluded that the second position is much more probable for the interaction between the synthetized compound and BSA (−33.6 vs. −29.0 kJ mol^−1^). The reason for the difference between these values lies in the fact that simulations were performed at 25 °C and that the BSA crystallographic structure might be partially different from the structure in the solution. Within the second active position, one conventional hydrogen bond is formed between **Me_3_SnL** and ARG194. Several van der Waals interactions were found with the surrounding amino acids (SER343, SER453, LEU210 and ASP450). Stacking interactions between aromatic parts of molecules are formed between the complex and TRP213. The rest of the interactions are very weak—for example, alkyl and π−alkyl. Again, the main contribution to the binding energy comes from weak interactions (−34.3 kJ mol^−1^, Table S7). Based on all of these results, it can be concluded that the number of weak interactions is the main reason for the higher spontaneity of the binding process and that the theoretical results reproduce the spectrofluorometric experiments well. A similar position including ARG194, ARG198, ARG217 and TRP213 was found as the preferential binding position for naproxen, a nonsteroidal anti-inflammatory drug, and other compounds [53].

### 2.4. In Vitro Screening

To assess cell viability, a diverse concentration range of **Me_3_SnL** was applied to all cell lines (MCF-7, A375, HCT116, 4T1, B16 and CT26) for 72 h. Subsequently, MTT and CV assays were conducted following the incubation period. The results demonstrated a reduction in cell viability across all cell lines, with IC_50_ values falling within the micromolar range (Table 2, Figure S7). The activity of **Me_3_SnL** against the investigated tumor cell lines was found to be superior to that of **HL**. On the other hand, as expected, **Me_3_SnL** was found to be less active than the appropriate diphenyltin(IV) compound (**Ph_2_SnL_2_**) [27]. Less effective than cisplatin, **Me_3_SnL** was found on the 4T1 (7.6 times, CV assay) and CT26 (3.4 times, CV assay) cell lines. With a comparable activity (CV assay) to that of cisplatin, **Me_3_SnL** was exhibited on the MCF-7 and A375 cell lines, while a more efficient activity was found against the HCT116 (1.5 times) and B16 (1.7 times) cell lines. To evaluate the selectivity of the tested compound toward malignant phenotypes, human embryonic fibroblasts (MRC5) were used (Figure S8). According to the results shown in Table 2, **Me_3_SnL** exhibited high selectivity, with the following selectivity indices (SI): B16 (SI = 8.9), 4T1 (SI = 5), CT26 (SI = 5.8), A375 (SI = 8.6), MCF7 (SI = 8.2), and HCT116 (SI = 11.7).

### 2.5. Mechanism of Action

**Figure 6:**
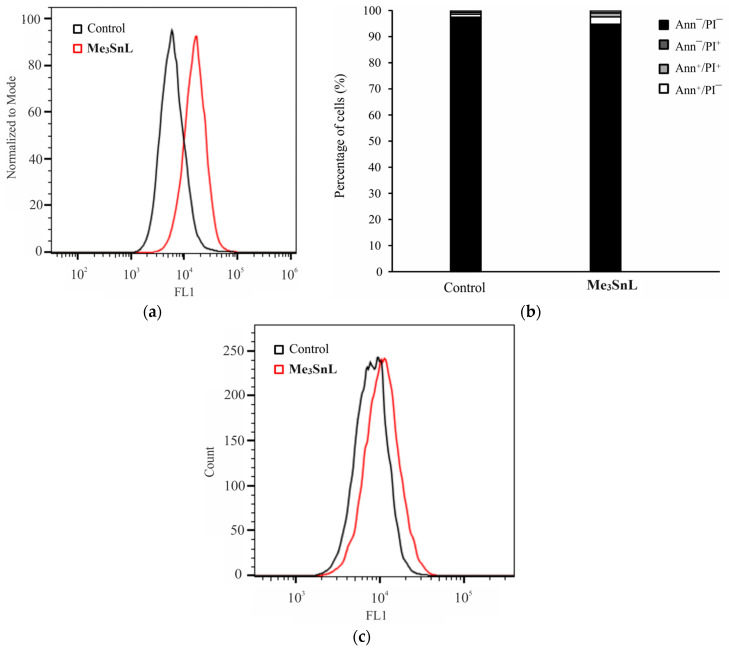
Evaluation of the mechanism of action during the treatment with **Me_3_SnL**. A375 cells underwent exposure to the IC_50_ concentration of **Me_3_SnL** for 48 h, followed by flow cytometry analysis. (**a**) CFSE staining; (**b**) Ann/PI staining; (**c**) ApoStat staining.

To elucidate the precise mechanism of action, A375 cells were subjected to an IC_50_ concentration of **Me_3_SnL** and subsequently analyzed using flow cytometry. A strong inhibition of cell proliferation was observed after the treatment with **Me_3_SnL** for 48 h (Figure 6a). Ann/PI double staining showed no apoptotic cells after the treatment (Figure 6b). However, slight caspase activation was observed (Figure 6c).

**Figure 7:**
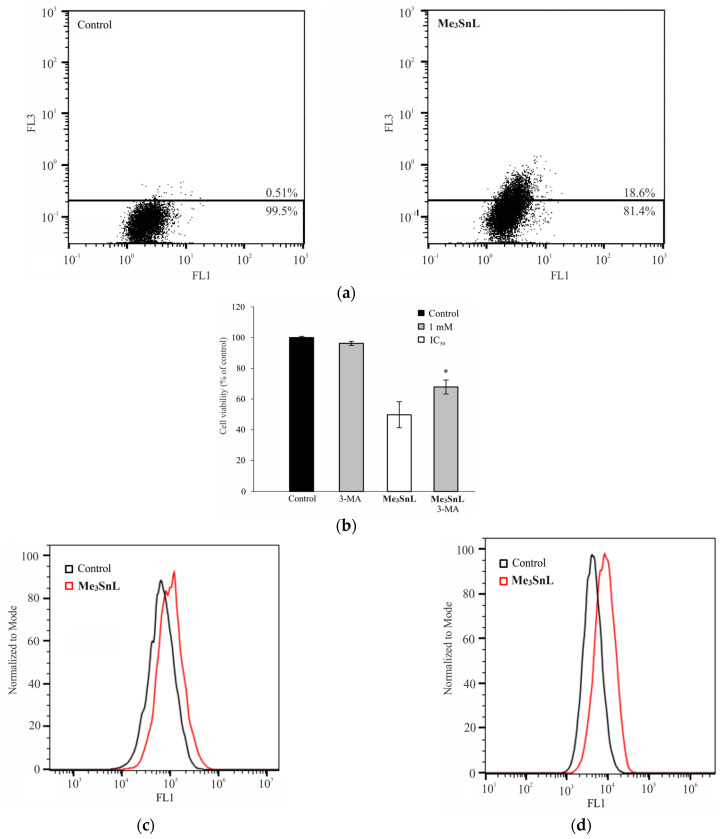
Evaluation of the mechanism of action during the treatment with **Me_3_SnL**. A375 cells underwent exposure to the IC_50_ concentration of **Me_3_SnL** for 48 h, followed by flow cytometry analysis. (**a**) Acridine orange staining; (**b**) cell viability determination in concomitant treatment with 3-MA by a CV assay. * *p* < 0.05. The effect of **Me_3_SnL** on the production of ROS/RNS and lipid peroxidation: (**c**) A375 cells were prestained with DHR 123 staining and exposed to an IC_50_ concentration of **Me_3_SnL** for 48 h, followed by flow cytometry; (**d**) after 48 h of treatment with **Me_3_SnL**, A375 cells were stained with BODIPY, followed by flow cytometry.

In parallel, the treatment potentiated autophagosome formation (Figure 7a), while the combined treatment with the autophagy inhibitor 3-MA showed a recovery of cell viability, pointing out the cytodestructive role of this process (Figure 7b). Our previous finding indicated that diphenyltin(IV) complexes with carboxylato *N*-functionalized 2-quinolone ligands inhibited proliferation and triggered caspase-dependent apoptosis in HCT116 cells. It is evident that Me instead of Ph moieties bonded to tin(IV) completely converted the activity of Ph_2_SnL_2_ complexes from the induction of apoptosis to autophagic cell death [27].

To evaluate the effect of the applied treatment on the production of reactive oxygen and nitrogen species (ROS/RNS) and lipid peroxidation, DHR 123 and BODIPY staining were used, respectively. As presented in Figure 7, an increased production of ROS/RNS as well as lipid peroxidation was observed. A straightforward connection between lipid peroxidation and autophagy is described in the literature [54]. Namely, products of lipid peroxidation such as unsaturated lipid peroxidation aldehydes promoted autophagy [55,56]. For healthy tissue maintenance, autophagy is essential for recycling damaged cellular structures. However, in the context of cancer treatment, robust lipid peroxidation leads to autophagic cell death, as in the case of **Me_3_SnL**.

## 3. Materials and Methods

### 3.1. General Remarks

The ligand precursor, 3-(4-methyl-2-oxoquinolin-1(2H)-yl)propanoic acid (**HL**), was prepared by the procedure described in the literature [27]. Trimethyltin(IV) chloride was obtained (Sigma-Aldrich Taufkirchen, Germany), methanol (Carl Roth, Karlsruhe, Germany) and *n*-hexane (Carl Roth) were obtained commercially and used without purification and the toluene (Carl Roth) was kept over the molecular sieves. Deutero solvents were purchased from Deutero Gmbh. Elemental analysis was carried out by the University of Belgrade (Vario EL III C, H, N and S Elemental Analyzer), and the experimentally found values are given in the experimental part. The low infrared spectrum was recorded by a Bruker Vertex 70 with a Diamond ATR unit (range of 4000 to 200 cm^−1^). The ^1^H, ^13^C and ^119^Sn NMR of the complex were recorded in deuterated CDCl_3_ solvent on a Bruker AvanceTM 400 MHz Spectrometer (Karlsruhe, Germany; ^1^H, 400.23 MHz; ^13^C, 100.23 MHz; ^119^Sn NMR, 149.25 MHz). To determine the lipophilicity and stability of the complex, the UV/Vis spectra were recorded on a Shimadzu double-beam spectrophotometer, in a wavelength range from 200 to 500 nm at room temperature. For stability, the measurement of the UV/Vis spectra was repeated after 24, 48 and 72 h.

### 3.2. Synthesis of Me_3_SnL

The complex 3-(4-methyl-2-oxoquinolin-1(2H)-yl)propanoato)trimethyltin(IV) (**Me_3_SnL**) was synthesized as follows: to a suspension of the ligand precursor, 0.5 mmol of **HL** (115.62 mg) in 5 mL of methanol, a solid KOH (0.5 mmol) was added, and after stirring for 2 h at room temperature, a clear solution was formed. The methanol was removed under reduced pressure to dryness. Then, 0.5 mmol of Me_3_SnCl (99.63 mg) dissolved in 5 mL of toluene was added, and the solution was stirred overnight (12 h, Scheme 1). The solution was filtrated to remove KCl and evaporated to dryness under reduced pressure.

(3-(4-methyl-2-oxoquinolin-1(2H)-yl)propanoato)trimethyltin(IV), **Me_3_SnL**: colorless sticky solid; Yield: 65%; Anal. calcd. for (C_16_H_21_NO_3_Sn): C, 48.77; H, 5.37; N, 3.55; Found C, 48,49; H, 5.44; N, 3.50; Selected FT-IR data (ATR) cm^−1^: 2956, 2920, 2852, 2361, 2338, 1637, 1574, 1498, 1453, 1389, 1371, 1236, 1098, 866, 779, 749, 694, 665, 594, 546, 501, 447, 413; ^1^H NMR (CDCl_3_, ppm): *δ* 7.72 (1H, dd, H^5^); *δ* 7.57 (1H, ddd, H^6^); *δ* 7.48 (1H, d, H^8^); *δ* 7.26 (1H, m, H^7^); *δ* 6.58 (1H, m, H^3^); *δ* 4.57 (2H, m, H^13^); *δ* 2.73 (2H, m, H^12^); *δ* 2.46 (3H, m, H^14^); *δ* 0.58 (9H, s, H^α^; ^2^*J*(^119^Sn, ^1^H) = 56 Hz); ^13^C NMR (CDCl_3_, ppm): δ −2.75 (C^α^; ^1^*J*(^119^Sn, ^13^C) = 400/384 Hz); δ 18.49 (C^14^); δ 32.23 (C^12^); *δ* 38.16 (C^13^); *δ* 113.79 (C^8^); *δ* 120.34 (C^3^); *δ* 121.13 (C^10^); *δ* 121.39 (C^6^); *δ* 124.9 (C^5^); *δ* 130.05 (C^7^); *δ* 138.13 (C^9^); *δ* 146.19 (C^4^); *δ* 161.19 (C^2^); *δ* 175.52 (C^11^); ^119^Sn NMR (CDCl_3_, ppm): 132.90. ^119^Sn NMR (DMSO-d_6_, ppm): −106.27.

### 3.3. Crystal Structure Determination and Refinement

Data for **HL** were obtained at 100 K using a Bruker Venture D8 diffractometer. The structure was solved through direct methods and subsequently refined using full-matrix least-squares procedures on *F*^2^ with SHELXS/SHELXL-2013 [57]. Anisotropic refinement was applied to all non-hydrogen atoms, while C-bonded hydrogen atoms were refined using a riding model. The position of the O-bonded hydrogen atom was taken from difference Fourier maps and freely refined. The complete datasets are available at CCDC under the accession number 2323674 and are available through https://www.ccdc.cam.ac.uk/structures/ accessed on 6 January 2024. Selected crystal and structural refinement data of **HL** are given in Table S1.

### 3.4. Computational Methods

#### 3.4.1. Hirshfeld Surface Analysis

The stability of the crystallographic structure is determined by the presence of various intermolecular interactions. One way of investigating them is through Hirshfeld surface analysis performed on the crystallographic structure. CrystalExplorer [58] was applied to identify and quantify the most important contacts that stabilize the crystal structure of **HL**. The Hirshfeld surface is presented by a graph connecting distances between the two nearest nuclei (de) and the distance between the nuclei and external surface (di) [59,60]. These distances are normalized and colored red, white and blue if the separation is shorter, equal or longer than the van der Waals radii of interacting atoms. In this contribution, the normalized distances are between −0.7316 (red) and 1.1187 (blue). Fingerprint plots show the distribution and percentages of specific interactions presented in the Supplementary Material.

#### 3.4.2. Structure Optimization and Spectral Characterization

The optimizations of the ligand and complex structures were performed in the Gaussian 09 Program Package [61]. The starting crystallographic structure of the ligand was optimized by employing the Global Hybrid Generalized Gradient Approximation (GGA) functional B3LYP-D3BJ [62,63] in conjunction with the 6-31++G(d,p) [64] basis set. On the other hand, for obtaining the most stable structure of the complex, the same functional and basis set for H, C, N and O atoms were used, while Def2-TZVP was applied for Sn [65]. The D3BJ correction of the B3LYP functional was selected to encounter non-covalent interactions that additionally stabilize structures. The same level of theory was previously successfully employed for the structural and spectral investigation of other Sn-containing compounds [34]. The optimizations were performed without any geometrical constraints, and the minima on the potential energy surface were verified by the absence of imaginary frequencies. The intramolecular interactions were identified through the Natural Bond Orbital (NBO) analysis [66,67]. The solvent effect on spectra was examined by applying the Conductor-like Polarizable Continuum (CPCM) solvent model [68]. The NMR spectra were predicted for the ligand and complex by the Gauge Independent Atomic Orbital (GIAO) approach [69,70].

### 3.5. Lipophilicity Assay

The lipophilic character of the trimethyltin(IV) complex was determined by a flask-shaking method [71]. A stock solution of the compound **Me_3_SnL** in DMSO (10^−3^ M) was prepared, and then a calibration curve was recorded for solutions at the concentrations of 6.62 × 10^−6^, 1.31 × 10^−5^, 1.96 × 10^−5^, 2.60 × 10^−5^, 3.22 × 10^−5^, 3.84 × 10^−5^, 4.76 × 10^−5^, 5.66 × 10^−5^, 6.54 × 10^−5^ and 7.12 × 10^−5^ M. A total of 1 mL of the stock solution was added to the water/*n*-octanol system, and the concentration of the compound was 10^−4^. The solution was then vortexed for 1 h at room temperature, and the two-phase solution was left for the next 24 h to separate the water and *n*-octanol phases. The concentrations of the compounds of *n*-octanol (c_o_) and the water (c_w_) phase were calculated by measuring absorbance using the previously recorded calibration graphic. The *LogP* value was calculated using the following equation:

*logP* = log(c_o_/c_w_) (3)

### 3.6. Spectrofluorimetric Investigation of the BSA Binding Affinity 

The binding process between Bovine Serum Albumin (BSA) and **Me_3_SnL** was examined by spectrofluorimetric titration on a Cary Eclipse MY2048CH03 instrument. The excitation wavelength was set to 295 nm, corresponding to the tryptophan and other fluorescent amino acid residues in the protein structure. The emission spectra were recorded between 310 and 500 nm. The scan rate was set to 600 nm min^−1^, and both slits were 5 nm. The concentration of BSA was held constant (5 × 10^−6^ M) in 1 M of phosphate buffer saline (pH = 7.4) [16]. The concentration of the complex changed in the range between 1 and 10 × 10^−6^ M. The emission spectra were recorded two minutes after the addition of the complex. The relative decrease in the fluorescent emission was followed, and the data were analyzed by the double-log Stern–Volmer quenching equation:

(4) $$\log\left(\frac{I_{0}-I}{I}\right)=\log K_{b}+n\log\left[Q\right]$$

In the previous Equation (4), *I*_0_ and *I* are the fluorescence intensities of BSA without and with the added complex, *K_b_* is the binding constant, *n* is the number of binding places and [*Q*] is the concentration of the metal complex. The thermodynamic parameters of binding were calculated from the Van’t Hoff’s plots following the measurements at three temperatures (27, 32 and 37 °C):

(5) $$\ln K_{b}=-\frac{∆H_{b}}{RT}+\frac{∆S_{b}}{R}$$

### 3.7. Molecular Docking

The molecular docking simulations were utilized to complement the spectrofluorometric measurements towards BSA. The Autodock 4.2 software [72] with the Lamarckian Genetic Algorithm was employed to determine the binding affinity of the complex towards the selected biomolecule [73,74]. The mentioned method included the following parameters: a maximum of 250,000 energy evaluations, 27,000 generations and mutation and crossover rates of 0.02 and 0.8, respectively. These simulations included the preparation of the ligand and protein and the grid formation. The optimized structure of **Me_3_SnL** from the previous section was used as a flexible ligand. The crystal structure of BSA was taken from the RCSB Protein Data Bank in PDB form (PDB ID: 4OR0) [53]. The initial structure preparation was performed in the BIOVIA Discovery Studio 4.0 [75]. This included a removal of chain B, residual atoms, heteroatoms and water molecules. The AutoDockTools graphical interface [76] was employed to calculate the Kollman partial charges and add polar hydrogen atoms. The search space was restricted to a grid box with dimensions of 60.0 × 60.0 × 60.0 Å^3^ and a spacing of 0.375 Å. The grid box was concentrated around two active positions of BSA with the following XYZ coordinates: 15.0, 35.0 and 89.0 Å in the vicinity of TRP134 and −6.0, 21.0 and 106 Å in the vicinity of TRP213. The calculated binding affinity between the complex and BSA contains several contributions:

Δ**G**_bind_ = Δ**G**_vdw+hbond+desolv_ + Δ**G**_elec_ + Δ**G**_total_ + Δ**G**_tor_ − Δ**G**_unb_ (6)

In the previous Equation (6), the ΔG_bind_ is the estimated free energy of binding, the first contribution includes energies of dispersion and repulsion, hydrogen bonds and desolvation, the second represents the electrostatic energy, ΔG_total_ is the total internal energy, ΔG_tor_ is the torsional free energy and ΔG_unb_ is the unbound system’s energy.

### 3.8. In Vitro Studies

#### 3.8.1. Reagents and Cells

The reagents and cells used in this study were sourced from various manufacturers as follows: the culture medium RPMI-1640 and fetal bovine serum (FBS) were obtained from Capricorn Scientific GmbH (Hessen, Germany). 3-(4,5-dimethythiazol-2-yl)-2,5-diphenyltetrazolium bromide (MTT) was purchased from AppliChem (MO, USA). The Penicillin Streptomycin solution was acquired from Biological Industries (Cromwell, CT, USA). Crystal violet (CV), phosphate-buffered saline (PBS), dimethyl sulfoxide (DMSO), carboxyfluoresceindiacetate succinimidyl ester (CFSE) and 3-methyl adenine (3-MA) were obtained from Sigma (St. Louis, MO, USA). Paraformaldehyde (PFA) was supplied by Serva (Heidelberg, Germany). Annexin V-FITC (AnnV) was procured from BD (Pharmingen, San Diego, CA, USA). ApoStat was obtained from R&D Systems (Minneapolis, MN, USA). C11 BODIPY 581/591 was obtained from Cayman Chemical (Ann Arbor, MI, USA). Dihydrorhodamine 123 (DHR 123) was purchased from Thermo Fisher Scientific (Waltham, MA, USA). The cell lines, including human breast adenocarcinoma (MCF-7), human colorectal carcinoma (HCT116), human melanoma (A375), mouse breast carcinoma (4T1), mouse colon carcinoma (CT26), mouse melanoma (B16) and human embryonic fibroblasts (MRC5), were sourced from the American Type Culture Collection (ATCC, Manassas, VA, USA).

All cell lines, namely, MCF-7, HCT116, A375, 4T1, CT26, B16 and MRC5, were cultured in HEPES-buffered RPMI-1640 medium. The culture medium was supplemented with 10% heat-inactivated FBS and antibiotics (100 units/mL penicillin/100 μg/mL streptomycin). The cells were maintained under standard conditions at 37 °C in a humidified atmosphere with 5% CO_2_. For viability assessments in 96-well plates, the cell lines were seeded at the following densities: 4 × 10^3^ cells/well for 4T1, 5 × 10^3^ cells/well for HCT116 and A375, 3 × 10^3^ cells/well for B16 and 8 × 10^3^ cells/well for MCF-7 and MRC5. For flow cytometric analyses in six-well plates, the density of A375 cells was set at 1.5 × 10^5^ cells/well.

#### 3.8.2. Determination of Cell Viability (MTT and CV Assays)

All cell lines were seeded overnight and exposed to **Me_3_SnL**. Following 72 h of incubation, the supernatant was removed, and the cells were washed with PBS. Subsequently, the cells were incubated with an MTT solution at a final concentration of 0.5 mg/mL, and incubation at 37 °C continued until purple formazan crystals developed. After discarding the formed dye, DMSO was added to dissolve the formazan crystals. Absorbance was measured at 540 nm/670 nm, and the results were expressed as a percentage of the control value, which was arbitrarily set to 100%.

For the CV assay, post-incubation, the cells were washed with PBS and fixed with 4% paraformaldehyde (PFA) at room temperature (RT) for 10 min. Subsequently, the cells were stained with a 1% CV solution for 20 min, followed by washing in tap water and air drying. The dye was dissolved in 33% acetic acid, and absorbance was measured at 540 nm/670 nm. The results were expressed as a percentage of the control value, set arbitrarily at 100%.

To elucidate the nature of the detected autophagy, a concurrent treatment involving **Me_3_SnL** and the autophagy inhibitor 3-MA was conducted. A375 cells were simultaneously exposed to the IC_50_ concentration of **Me_3_SnL** and a concentration of 1 mM 3-MA. Cell viability was assessed after 72 h using the CV assay.

#### 3.8.3. Annexin V/Propidium Iodide (PI)

To detect apoptosis, A375 cells were treated with **Me_3_SnL** (IC_50_ concentration) for 48 h. Following the incubation period, the cells were rinsed with PBS and then stained with Annexin V and PI (both at a final concentration of 15 μg/mL) for 15 min at RT, protected from light. Subsequently, the cells were suspended in Annexin V-binding buffer (ABB) and analyzed using flow cytometry (CytoFLEX Flow Cytometer, Beckman Coulter, Life Sciences, Indianapolis, IN, USA).

#### 3.8.4. ApoStat Staining

To assess caspase activation, A375 cells were treated with the pan-caspase inhibitor ApoStat. Following 30 min of incubation at 37 °C, the cells were washed with PBS and subjected to analysis using flow cytometry (CytoFLEX Flow Cytometer, Beckman Coulter, Life Sciences, Indianapolis, IN, USA).

#### 3.8.5. AO Staining

To identify the presence of autophagosomes, A375 cells were treated with AO solution (1 µg/mL) for 15 min at 37 °C. Subsequently, the cells were washed with PBS, resuspended and subjected to analysis using flow cytometry (CytoFLEX Flow Cytometer, Beckman Coulter, Life Sciences, Indianapolis, IN, USA).

#### 3.8.6. CFSE Staining

Before seeding, A375 cells underwent staining with CFSE (1 μM) for 10 min at 37 °C. Following the incubation period, the cells were washed, seeded and exposed to an IC_50_ concentration of **Me_3_SnL**. After 48 h of incubation, the cells were trypsinized, washed and resuspended in PBS. The final analysis was conducted using flow cytometry (CytoFLEX Flow Cytometer, Beckman Coulter, Life Sciences, Indianapolis, IN, USA).

#### 3.8.7. Measurement of Reactive Oxygen and Nitrogen Species (ROS/RNS) Generation

For the detection of ROS/RNS production, DHR 123 staining was employed. A375 cells were prestained with DHR 123 (1 µM) for 20 min at 37 °C, followed by treatment with an IC_50_ concentration of **Me_3_SnL**. After 48 h of incubation, the cells underwent washing, trypsinization and subsequent analysis using flow cytometry (CytoFLEX Flow Cytometer, Beckman Coulter, Life Sciences, Indianapolis, IN, USA).

#### 3.8.8. Detection of Lipid Peroxidation

For the detection of lipid peroxidation, A375 cells were seeded overnight and treated with an IC_50_ concentration of **Me_3_SnL** for 48 h. After the incubation period, the cells were stained with BODIPY to a final concentration of 2 µM for 30 min. Finally, the cells were washed with PBS, resuspended and analyzed using flow cytometry (CytoFLEX Flow Cytometer, Beckman Coulter, Life Sciences, Indianapolis, IN, USA).

#### 3.8.9. Statistical Analysis

The experiments detailed in the paper were repeated independently three times, and the data are expressed as the mean ± SD. The significance between the groups was assessed using the Student *t*-test, with two-sided *p* values below 0.05 considered statistically significant.

## Figures and Tables

**Table 1:** Experimental and theoretical (at B3LYP-D3BJ/6-311++G(d,p) level of theory) ^1^H and ^13^C NMR chemical shifts of **Me_3_SnL**.

^1^H			^13^C		
H atom	Exp. [ppm]	Calc. [ppm]	C atom	Exp. [ppm]	Calc. [ppm]
C^15,16,17^–H	0.58	−0.21	C^15,16,17^	−2.75	−0.39
C^14^–H	2.46	2.36	C^14^	18.49	22.09
C^12^–H	2.73	2.55	C^12^	32.23	36.17
C^13^–H	4.57	4.34	C^13^	38.16	40.91
C^3^–H	6.58	6.45	C^8^	113.79	112.64
C^6^–H	7.26	7.31	C^6^	120.34	118.93
C^8^–H	7.48	7.31	C^3^	121.13	120.47
C^5^–H	7.57	7.59	C^10^	121.39	124.67
C^7^–H	7.72	7.64	C^5^	124.90	124.68
R		0.998	C^7^	130.05	130.54
MAE [ppm]		0.19	C^9^	138.13	140.23
			C^4^	146.9	148.18
			C^2^	161.19	157.36
			C^11^	175.52	174.34
			R		0.999
			MAE [ppm]		2.02

**Table 2:** IC_50_ concentrations [µM] * determined after 72 h of action of **Me_3_SnL**, the ligand precursor (**HL**), **Ph_2_SnL_2_** and cisplatin are included for comparison [27].

Compound	Assays	MCF-7	A375	HCT116	4T1	B16	CT26	MRC5
**HL** **	MTT [µM]	>200						n.d.***
	CV [µM]	>200						n.d.
**Me_3_SnL**	MTT [µM]	5.7 ± 0.2	5.4 ± 0.5	6.2 ± 0.4	17.2 ± 2.4	8.1 ± 0.15	11.9 ± 1.5	/
	CV [µM]	11.4 ± 2.7	10.9 ± 1.9	8.0 ± 0.4	18.9 ± 1.8	10.6 ± 1.0	16.1 ± 2.0	93.9 ± 2.2
**Ph_2_SnL_2_** **	MTT [µM]	0.10 ± 0.00	0.30 ± 0.05	0.15 ± 0.02	0.30 ± 0.03	0.36 ± 0.01	0.30 ± 0.01	n.d.
	CV [µM]	0.20 ± 0.02	0.36 ± 0.05	0.26 ± 0.02	0.40 ± 0.01	0.39 ± 0.07	0.40 ± 0.07	n.d.
**Cisplatin** **	MTT [µM]	5.00 ± 0.70	8.10 ± 0.70	5.00 ± 2.30	1.60 ± 0.20	8.30 ± 1.60	4.30 ± 0.20	n.d.
	CV [µM]	12.60 ± 2.50	10.70 ± 1.60	12.30 ± 3.50	2.50 ± 0.10	18.00 ± 1.80	4.70 ± 0.40	n.d.

* mean ± SD; ** [27]; *** n.d.—not determined.

## Data Availability

Data supporting the obtained results can be obtained from the authors upon request.

## Supplementary Materials

The following supporting information can be downloaded at: https://www.mdpi.com/article/10.3390/ph17030372/s1, Crystal data and structure refinement for **HL** (Table S1); Bond lengths and angles for the compound **HL** (Table S2); Crystallographic and optimized (at B3LYP/6-311++G(d,p) level of theory) bond lengths (in Å) of **HL** (Table S3); Crystallographic and optimized (at B3LYP/6-311++G(d,p) level of theory) bond angles (in °) of **HL** (Table S4); The most important stabilization interactions (in kJ mol^−1^) of **HL** (Table S5); Assembling of the **HL** molecules through π-σ and π-π -stacking interactions (Figure S1); Fingerprint plots for the most numerous interactions within the crystal structure of **HL** (Figure S2); The most important stabilization interactions (in kJ mol^−1^) of **Me_3_SnL** (Table S6); FT-IR spectra of the trimethyltin(IV) complex **Me_3_SnL** (Figure S3); FT-IR spectra of the free ligand precursor (Figure S4); NMR spectra of the trimethyltin(IV) complex **Me_3_SnL**: (a) ^1^H; (b) ^13^C; (c) ^119^Sn (Figure S5); UV-Vis spectra of the trimethyltin(IV) complex in a water/DMSO solution, immediately after dissolution and after 24, 48 and 72 h (Figure S6); The important thermodynamic parameters for the best docking conformation of the investigated complexes with BSA (PDB ID:4OR0) (Table S7); Cell viability (Figures S7 and S8).
