# Supplementary material for: Trimethyltin(IV) Bearing 3-(4-Methyl-2-oxoquinolin-1(2H)-yl)propanoate Causes Lipid Peroxidation-Mediated Autophagic Cell Death in Human Melanoma A375 Cells
Source: Pharmaceuticals (Basel). 2024 Mar 14;17(3):372. doi: 10.3390/ph17030372 (PMC10975498; doi:10.3390/ph17030372)
Supplement: Supplementary file 1 [file pharmaceuticals-17-00372-s001.zip › pharmaceuticals-2837213-SI.pdf]

Supplementary material for

Trimethyltin(IV) Bearing 3-(4-Methyl-2-oxoquinolin-1(2H)-yl)propanoate Causes Lipid Peroxidation-Mediated Autophagic Cell Death in Human Melanoma A375 Cells

Marijana P. Kasalović [1,2], Dušan Dimić [3], Sanja Jelača [4], Danijela Maksimović-Ivanić [4], Sanja Mijatović [4], Bojana B. Zmejkovski [5], Simon H. F. Schreiner [6], Tobias Rüffer [6], Nebojša Đ. Pantelić [1,7,]* and Goran N. Kaluđerović [1,]*

[1] Department of Engineering and Natural Sciences, University of Applied Sciences Merseburg, Eber-hard-Leibnitz-Straße 2, 06217 Merseburg, Germany; marijana.kasalovic@pmf.kg.ac.rs

[2] Department of Chemistry, Faculty of Science, University of Kragujevac, Radoja Domanovića 12, 34000 Kragu-jevac, Serbia

[3] Faculty of Physical Chemistry, University of Belgrade, Studentski Trg 12–16, 11000 Belgrade, Serbia; ddimic@ffh.bg.ac.rs

[4] Department of Immunology, Institute for Biological Research ”Siniša Stanković” - National Institute of the Republic of Serbia, University of Belgrade, Bulevar despota Stefana 142, 11108 Belgrade, Serbia; sanja.jelaca@ibiss.bg.ac.rs (S.J.); nelamax@ibiss.bg.ac.rs (D.M.-I.); sanjamama@ibiss.bg.ac.rs (S.M.)

[5] Department of Chemistry, Institute of Chemistry, Technology and Metallurgy - National Institute of the Republic of Serbia, University of Belgrade, Studenski trg 12-16, 11000 Belgrade, Serbia; boja-na.zmejkovski@ihtm.bg.ac.rs

[6] Institute of Chemistry, Chemnitz University of Technology, Straße der Nationen 62, D-09111 Chemnitz, Ger-many; simon.schreiner@chemie.tu-chemnitz.de (S.H.F.S.); tobias.rueffer@chemie.tu-chemnitz.de (T.R.)

[7] Department of Chemistry and Biochemistry, Faculty of Agriculture, University of Belgrade, Nemanjina 6, 11080 Belgrade, Serbia

* Correspondence: pantelic@agrif.bg.ac.rs (N.Đ.P.); goran.kaluderovic@hs-merseburg.de (G.N.K.)

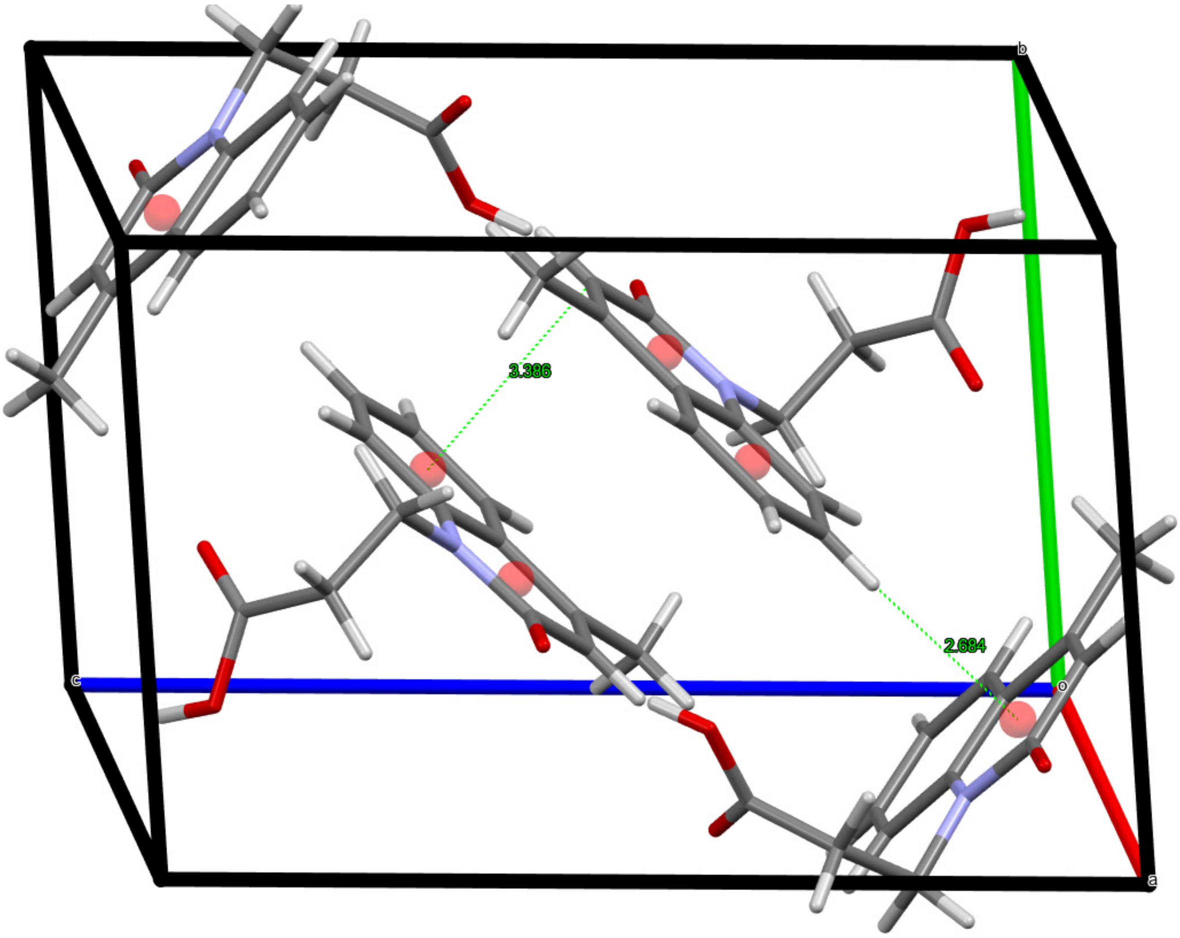

Figure S1. Assembling of the **HL** molecules through π-σ and π-π -stacking interactions (centroids are represented as red spheres).

Table S1. Crystal data and structure refinement for **HL**

**Empirical formula**	**HL ($C_{13}H_{13}NO_3$)**
**Formula weight**	231.24
**Temperature**	100 K
**Wavelength**	1.54178 Å
**Crystal system**	Monoclinic
**Space group**	$P2_1/c$
a	9.2211(7) Å
b	8.9635(8) Å
c	13.6375(11) Å
**α, β, γ (°)**	90, 109.678(2), 90
**Volume**	1061.36(15) $Å^3$
Z	4
**Calculated density**	1.447 mg/mm^3
**Absorption coefficient**	0.853 mm^{-1}
***F*(000)**	488
**Crystal size**	0.3 × 0.1 × 0.05 mm
**Radiation**	Cu Kα (λ = 1.54178 Å)
***θ* range for data collection**	10.188 to 138.266 °
**Limiting indices**	$-11 \leq h \leq 11$, $-10 \leq k \leq 9$, $-16 \leq l \leq 16$
**Reflections collected**	16651
**Independent reflections**	1978 [R_{int} = 0.0582, R_{sigma} = 0.0315]
**Data completeness**	99.9 %
**Absorption correction**	Multi-scan
**Max. and min. transmission**	0.7840 and 0.0.9586
**Refinement method**	Full-matrix least-squares on F^2
**Data / restraints / parameters**	1978/ 0 / 157
**Goodness-of-fit on F^2**	1.053
**Final *R* indices [$I \geq 2\sigma(I)$]**	R_1 = 0.0378, wR_2 = 0.0982
***R* indices (all data)**	R_1 = 0.0436, wR_2 = 0.1038
**Largest diff. peak and hole**	0.18 and -0.26 $e \cdot A^{-3}$

Table S2. Bond lengths and angles for the compound **HL**

Bond lengths		Bond angles (°)	
C2–O3	1.2498(17)	O3–C2–N1	119.65(12)
C2–N1	1.3806(17)	O3–C2–C3	122.54(12)
C2–C3	1.4405(18)	N1–C2–C3	117.80(11)
C3–C4	1.3476(19)	C4–C3–C2	122.68(12)
C4–C10	1.4447(18)	C3–C4–C10	118.94(12)
C4–C14	1.5034(17)	C3–C4–C14	120.63(12)
C5–C6	1.3780(19)	C10–C4–C14	120.42(12)
C5–C10	1.4093(18)	C6–C5–C10	121.24(12)
C6–C7	1.396(2)	C5–C6–C7	118.97(12)
C7–C8	1.3849(19)	C8–C7–C6	121.42(12)
C8–C9	1.4044(19)	C7–C8–C9	120.08(12)
C9–N1	1.4090(16)	C8–C9–N1	122.02(12)
C9–N10	1.4159(18)	C8–C9–C10	118.99(12)
C11–O1	1.2095(17)	N1–C9–C10	118.99(12)
C11–O2	1.3268(17)	C5–C10–C9	119.28(12)
C11–C12	1.5128(18)	C5–C10–C4	121.06(12)
C12–C13	1.5259(17)	C9–C10–C4	119.66(12)
C13–N1	1.4854(15)	O1–C11–O2	124.57(12)
		O1–C11–C12	123.00(12)
		O2–C11–C12	112.42(11)
		C11–C12–C13	113.07(11)

Table S3. Crystallographic and optimized (at B3LYP/6-311++G(d,p) level of theory) bond lengths (in Å) of **HL**.

Bond	Experimental	Optimized
N1–C2	1.3806(17)	1.404
C2–C3	1.4405(18)	1.448
C3–C4	1.3476(19)	1.353
C4–C14	1.5034(17)	1.504
C4–C10	1.4447(18)	1.448
C10–C9	1.4159(18)	1.421
C10–C5	1.4093(18)	1.407
C5–C6	1.3780(19)	1.382
C6–C7	1.396(2)	1.397
C7–C8	1.3849(19)	1.385
C8–C9	1.4044(19)	1.406
C9–N1	1.4090(16)	1.396
N1–C13	1.4854(15)	1.470
C13–C12	1.5259(17)	1.532
C12–C11	1.5128(18)	1.509
C11=O	1.2095(17)	1.208
C11–O	1.3268(17)	1.351
C2=O	1.2498(17)	1.229
R		0.99
MAE [Å]		0.008

Table S4. Crystallographic and optimized (at B3LYP/6-311++G(d,p) level of theory) bond angles (in °) of **HL**.

Angle	Experimental	Optimized
N1–C2–C3	117.80(11)	116.21
N1–C2–O	119.65(12)	120.32
C3–C2–O	122.54(12)	123.47
C3–C4–C14	120.63(12)	120.61
C3–C4–C10	118.94(12)	119.06
C14–C4–C10	120.42(12)	120.33
C4–C10–C5	121.06(12)	121.80
C4–C10–C9	119.66(12)	119.32
C10–C5–C6	121.24(12)	121.62
C5–C6–C7	118.97(12)	119.07
C6–C7–C8	121.42(12)	120.92
C7–C8–C9	120.08(12)	120.59
C8–C9–C10	118.99(12)	118.92
C8–C9–N1	118.99(12)	121.74
C9–N1–C2	121.87(11)	122.76
C9–N1–C13	122.56(11	122.82
C2–N1–C13	115.41(10)	114.39
N1–C13–C12	112.64(10)	112.85
C13–C12–C11	113.07(11)	113.66
C12–C11=O	123.00(12)	125.17
C12–C11–O	112.42(11)	112.44
O=C11–O	123.00(12)	122.37
R		0.97
MAE [°]		0.62

Table S5. The most important stabilization interactions (in kJ mol^{-1}) of **HL**.

Donor	Acceptor	Stabilization energy
π(C6−C7)	π*(C9−C8)	72
π(C6−C7)	π*(C5−C10)	94
π(C8−C9)	σ*(C4−C14)	75
π(C8−C9)	π*(C4−C10)	81
π(C8−C9)	π*(C6−C7)	98
π(C3−C4)	π*(C2−O)	101
π(C5−C10)	π*(C9−C8)	84
LP(N)	π*(C9−C8)	141
LP(N)	π*(C2−O)	247
LP(O)	π*(C11−O)	134
LP(O)	σ*(C11−C12)	63
LP(O)	σ*(N−C2)	100
LP(O)	σ*(C2−C3)	61

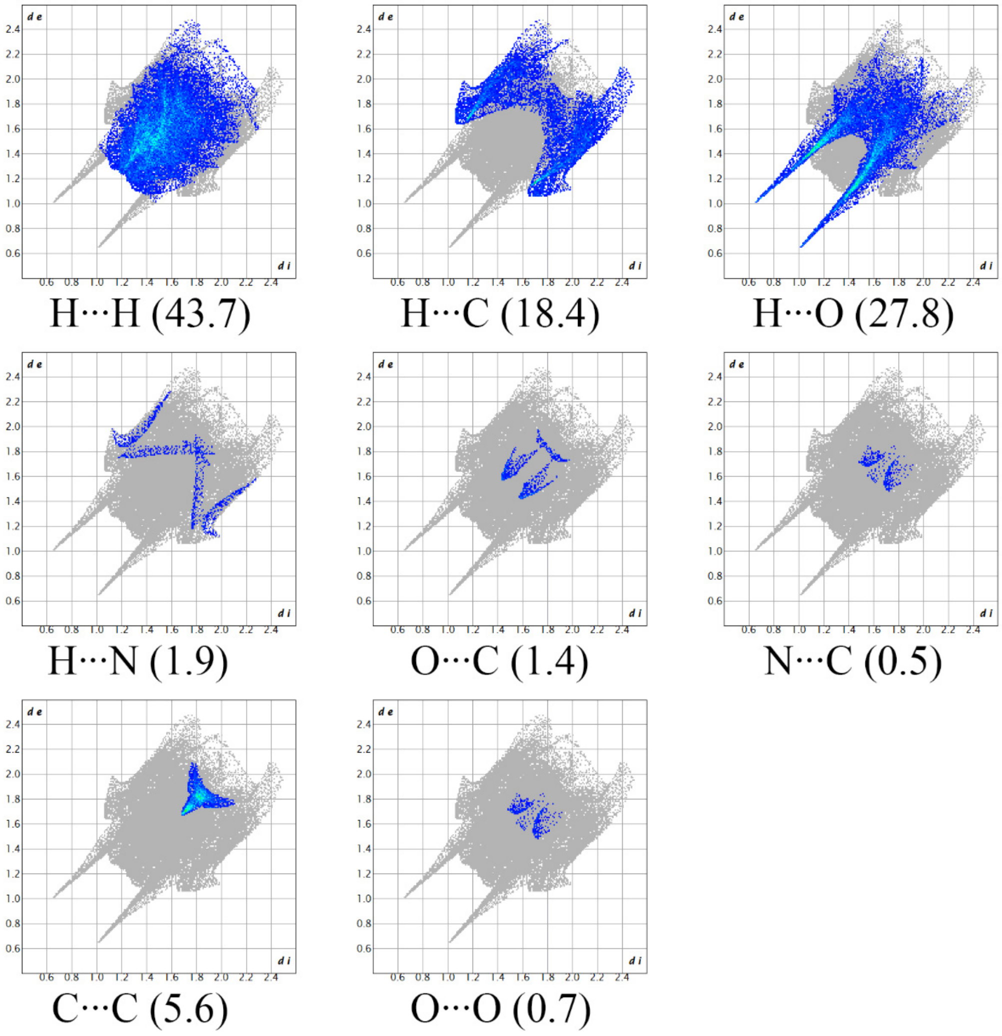

Figure S2. Fingerprint plots for the most numerous interactions within the crystal structure of **HL**.

Table S6. The most important stabilization interactions (in kJ mol^{-1}) of **Me_3SnL**.

Donor	Acceptor	Stabilization energy
π(C−O)	LP*(Sn)	79
π(C−O)	σ*(C−Sn)	43
π(C−O)	σ*(C−Sn)	291
LP(O)	LP*(Sn)	694
LP(O)	σ*(C−Sn)	46
σ(C−H)	LP*(Sn)	24
σ(C−H)	σ*(C−Sn)	67
σ(C−Sn)	σ*(C−Sn)	53

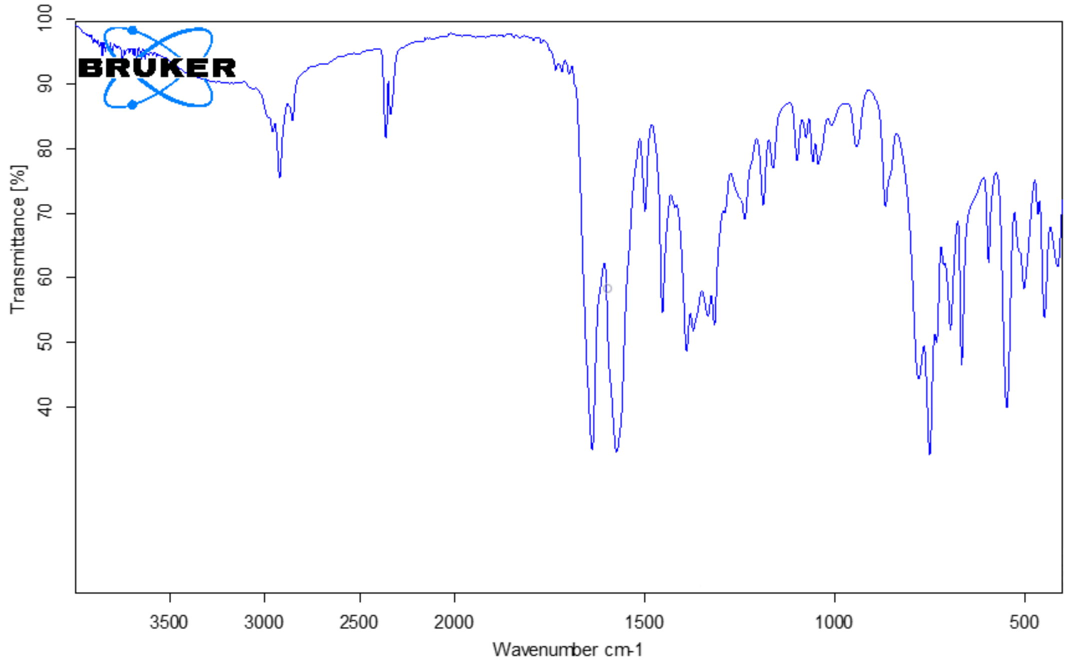

Figure S3. FT-IR spectrum of the trimethyltin(IV) complex **Me_3SnL.**

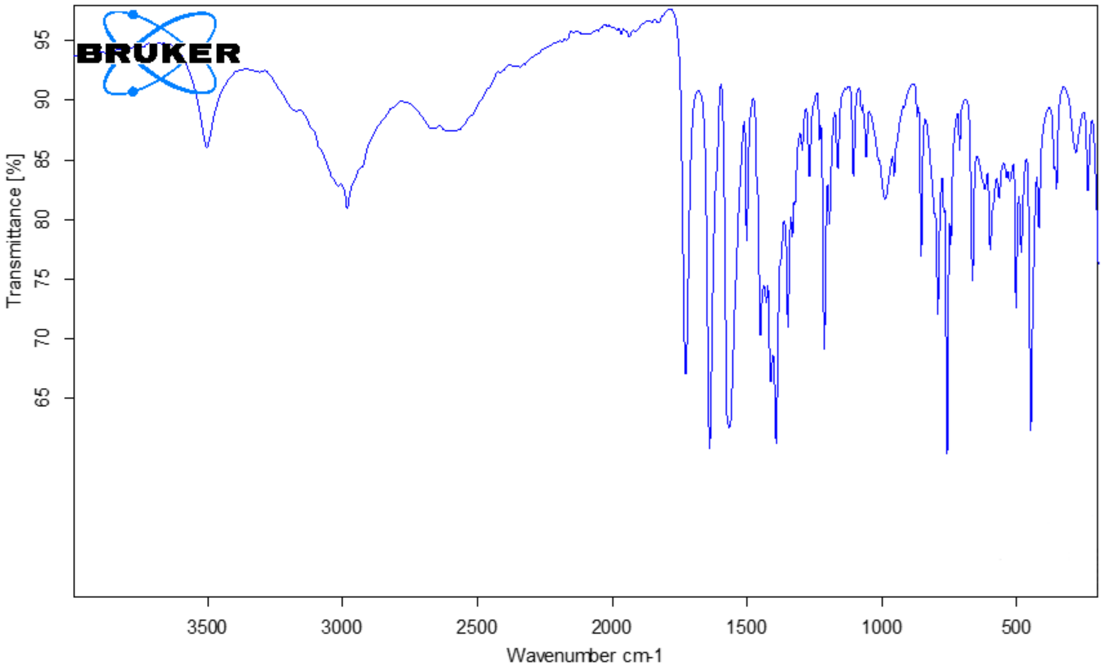

Figure S4. FT-IR spectrum of the ligand precursor (**HL**)

a)

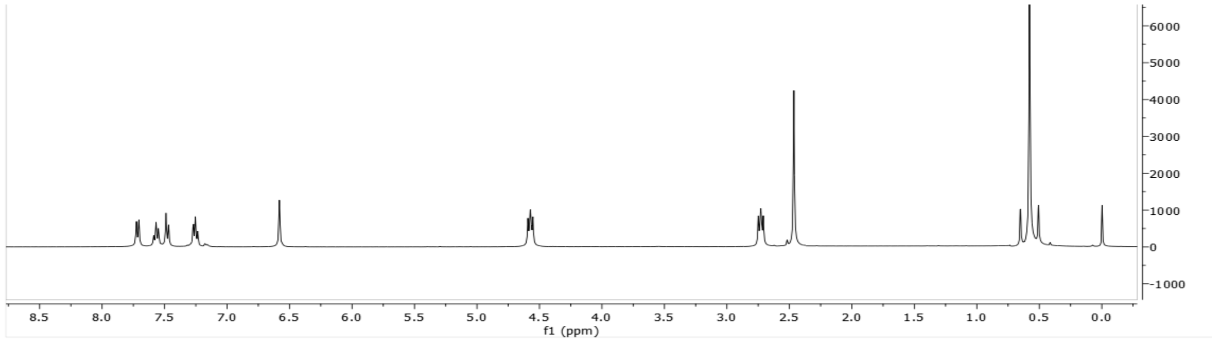

b)

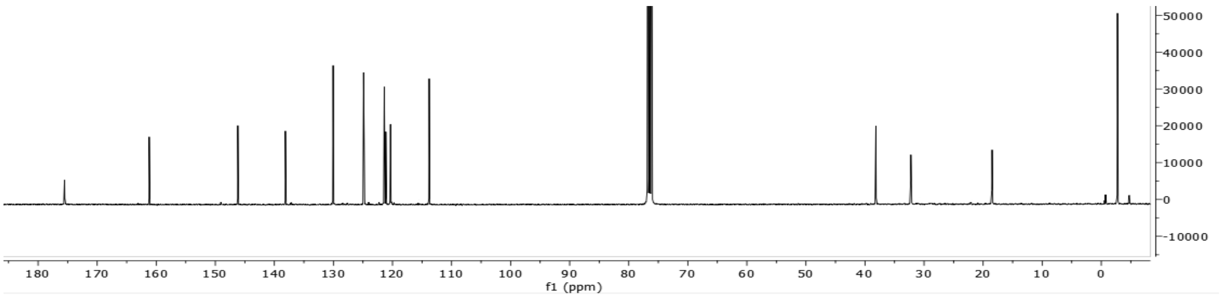

c)

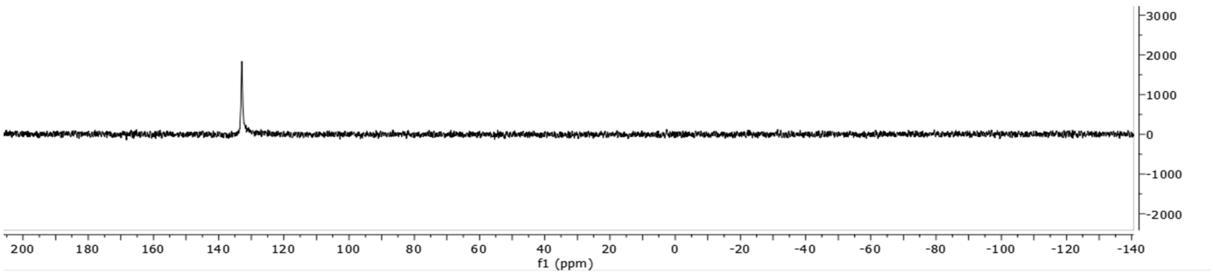

d)

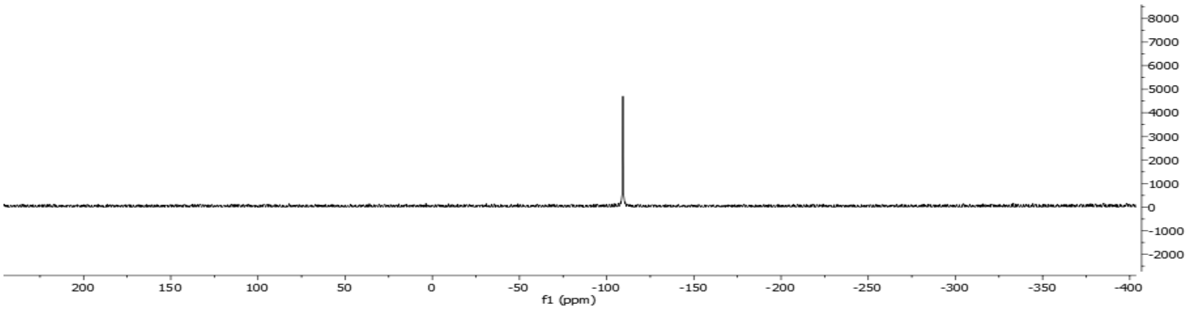

Figure S5. NMR spectra of the trimethyltin(IV) complex **Me_3SnL** (in $CDCl_3$) a) ^1H; b) ^{13}C; c) ^{119}Sn (in $CDCl_3$); d) ^{119}Sn (in DMSO-d_6).

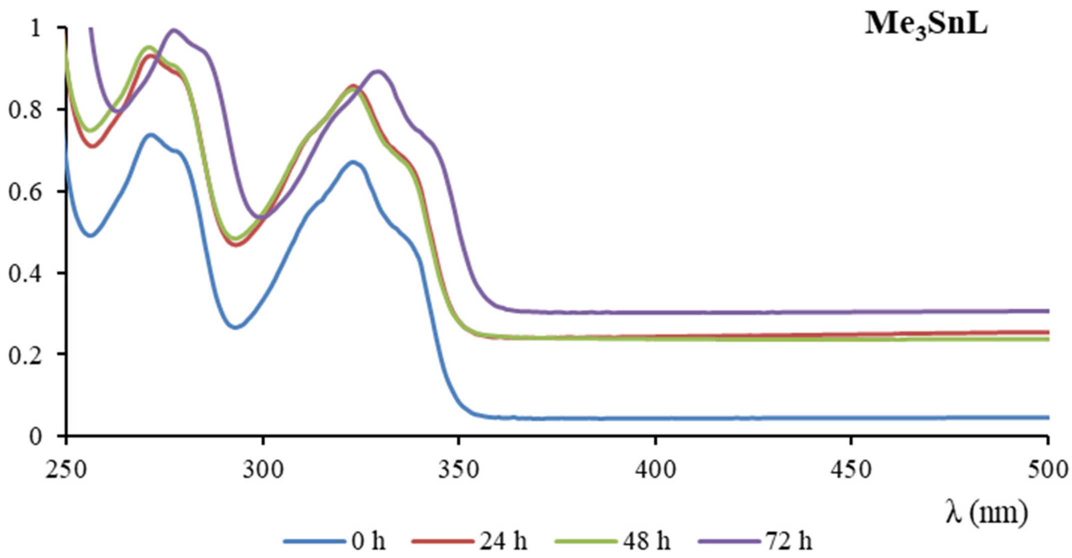

Figure S6. UV-Vis spectra of trimethyltin(IV) complex in water/DMSO solution, immediately after dissolution and after 24, 48 and 72 h.

Table S7. The important thermodynamic parameters for the best docking conformation of investigated complexes with BSA (PDB ID:4OR0).

**Active position**	Δ**G***bind*	**K**i (μM)	Δ**G***vdw+hbond+desolv*	Δ**G***elec*	Δ**G***total*	Δ**G***tor*	Δ**G***unb*
**TRP134**	–21.3	186.1	–27.8	0.2	–1.5	6.2	–1.5
**TRP213**	–29.0	8.34	–34.3	–0.9	–0.8	6.2	–0.8

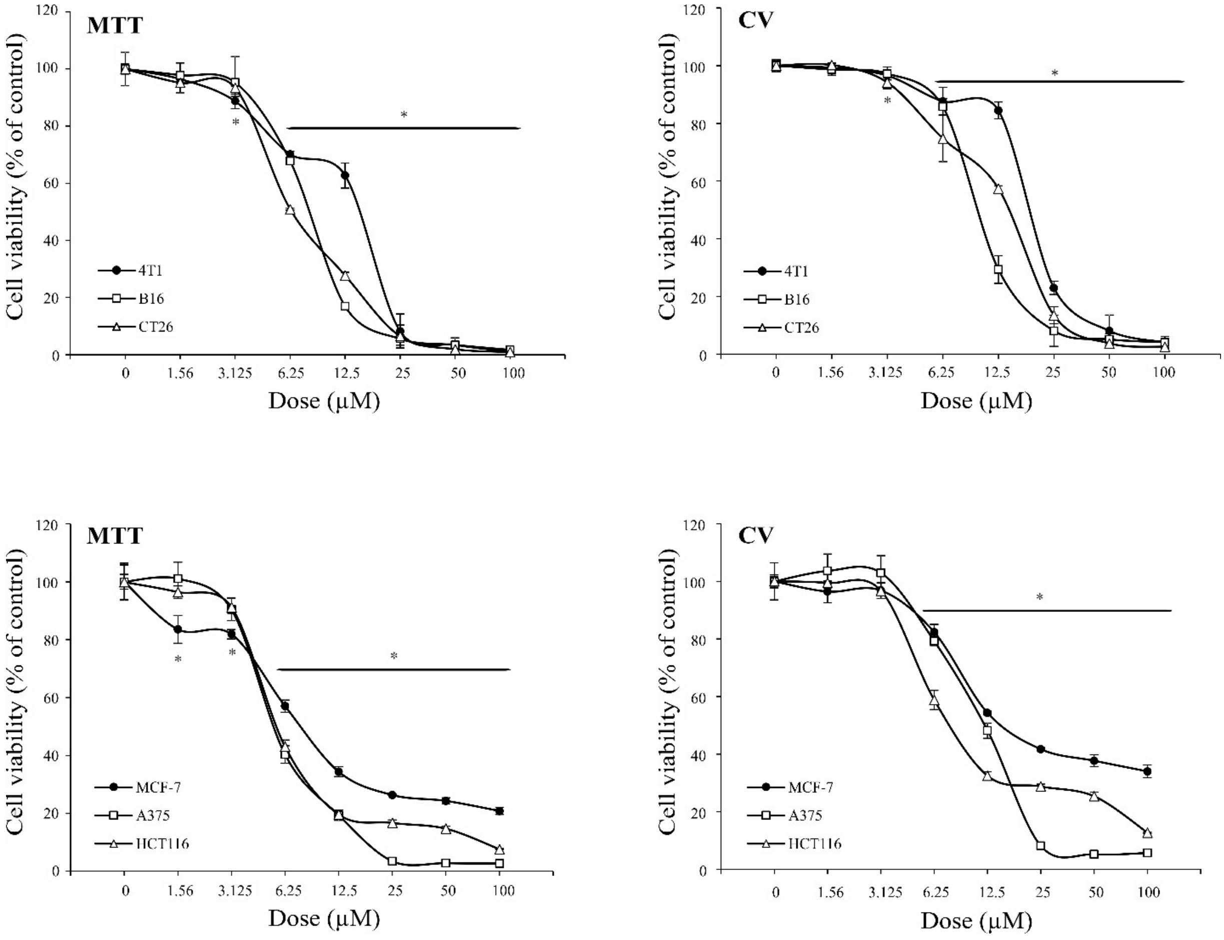

Figure S7. The impact of **Me3SnL** on the viability of both mouse and human cancer cell lines. Each cell line underwent a 72 h treatment with **Me3SnL**, followed by MTT and CV assays. The data depicted in the figure represents the mean ± SD of one representative experiment out of three independent and is presented as a percentage of untreated cells (with the untreated cells arbitrarily set as 100%). Statistical significance (*$p < 0.05$).

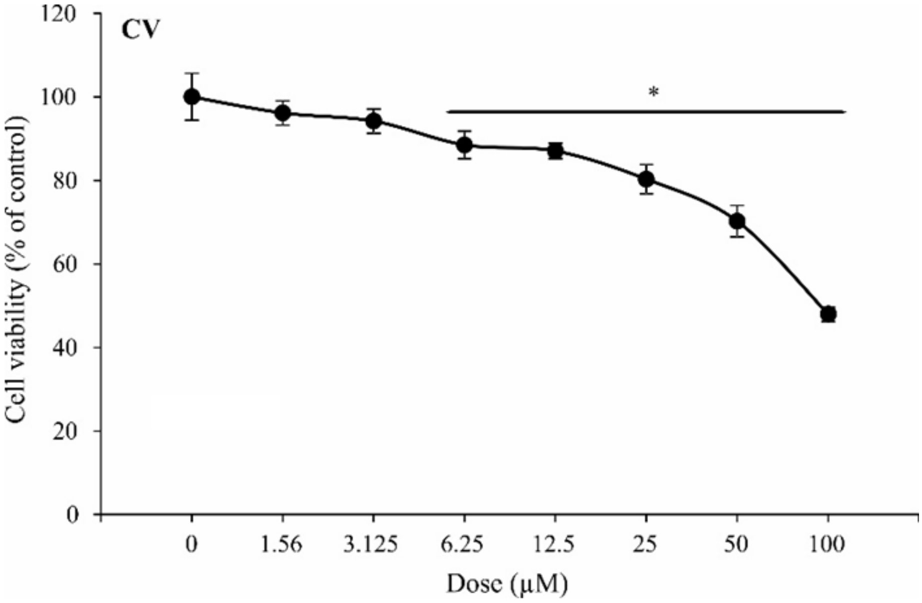

Figure S8. The impact of **Me_3SnL** on the viability of human embryonic fibroblasts (MRC5). Cells were treated with **Me_3SnL** for 72 h, and subsequent CV assay was conducted. The data illustrated in the figure represents the mean ± SD of one representative experiment out of three independent and is presented as a percentage of untreated cells (with untreated cells arbitrarily set as 100%). Statistical significance (*$p < 0.05$).
